# Eight Weeks of Inspiratory Muscle Training Improves Pulmonary Function in Disabled Swimmers—A Randomized Trial

**DOI:** 10.3390/ijerph16101747

**Published:** 2019-05-17

**Authors:** Paulina Okrzymowska, Monika Kurzaj, Wojciech Seidel, Krystyna Rożek-Piechura

**Affiliations:** 1 Department for Rehabilitation in Internal Medicine, University School of Physical Education, Al. I.J. Paderewskiego 35, Building P4, 51-612 Wrocław, Poland; monika.kurzaj@awf.wroc.pl (M.K.); krystyna.rozek-piechura@awf.wroc.pl (K.R.-P.); 2 Department of Paralympic Sports, University School of Physical Education, Al. I.J. Paderewskiego 35, Building P4, 51-612 Wrocław, Poland; wojciech.seidel@op.pl

**Keywords:** disabled swimmers, respiratory function, inspiratory muscle training

## Abstract

Background: According to the literature, inspiratory muscle fatigue may increase after swimming training (ST). This study aimed to examine the efficacy of 8-week inspiratory muscular training (IMT) in disabled swimmers, combined with standard sports training, on selected parameters of lung ventilation and the function of respiratory muscles. Methods: A total of 16 disabled swimming division athletes from Wroclaw’s ‘Start’ Regional Sports Association qualified for the study. The subjects were randomly divided into two groups (ST and IMT). Both groups participated in swimming training for 8 weeks (8 times a week). The IMT group additionally participated in inspiratory muscle training (8 weeks). In all respondents, a functional lung test and the respiratory muscle strength was measured. Results: After 8 weeks of training, a significant increase in ventilation parameters and respiratory muscle strength was observed only in the IMT group. In ST group 1, a 20% improvement in the strength of inspiratory muscles was achieved. Conclusions: The inclusion of IMT is an important element that complements swimming training, allowing for greater increases in lung ventilation parameters and the strength of respiratory muscles in disabled swimmers.

## 1. Introduction

Coaches of various sporting disciplines are increasingly introducing innovations in training processes, all aimed at improving their competitors’ achievements. One such innovation is strength training of the inspiratory muscles. According to the literature, exercise capacity and the strength of respiratory muscles is important to many athletes, especially competitive athletes [1,2,3,4]. The function of respiratory muscles, like all other skeletal muscles, improves in response to training. A reduction in the ability to generate strength in muscles or muscle groups is generally accepted as an indicator of the presence of fatigue. Also, the inspiratory muscles become tired after physical exercise [5,6]. Wüthrich et al. (2013) suggested that inspiratory muscle fatigue (IMF) may reduce weak exercise performance. The reason for this can be respiratory muscle metaboreflex. This phenomenon can impair blood flow to working lower limb muscles and accelerate the development of fatigue in these muscles [7]. It has been shown that exercise-induced inspiratory muscle fatigue occurs in response to 200 m race-paced swimming in all strokes [8]. Additionally, inspiratory muscle fatigue has been reported following 300 m and 400 m front crawl [9]. Interestingly, Brown and Kilding [10] suggest that that race distance while swimming does not substantially affect the degree of exercise-induced inspiratory muscle fatigue. At the same time, Jakovljevic and McConnell [11] showed that inspiratory muscle fatigue is greater when breathing frequency is reduced during high-intensity front crawl swimming. Lomax et al. (2013) demonstrated that inspiratory muscle fatigue is correlated with relative front crawl swimming velocity and stroke rate. Research has shown that inspiratory muscle fatigue occurred when swimming both at and above critical velocity for some of the test subjects [12]. According to these observations, it is worth using respiratory muscle training to improve respiratory muscle strength and endurance in swimmers [11].

Regular sports training of healthy people, and those who are patients, positively affects their physical and mental health, and has a positive effect on lung function, increasing expiratory volumes [13,14]. The use of additional IMT can, in fact, increase the strength of respiratory muscles, making it easier for swimmers to overcome hydraulic resistance and, thus, delay inspiratory muscle fatigue. Another advantage may be increased chest expansion, which results in increased expiratory lung volume [15].

The training of inspiratory muscles results in higher strength of the upper chest and neck muscles, which will improve the geometry of the chest (due to an increase in the vital capacity (VC) parameter). Increasing the thickness of the diaphragm after training also results in improved ventilation in the lungs [16].

A change in the muscle strength of swimmers resulting from swimming training can induce better inspiratory muscle function and increase lung function. Silvatti et al. (2012) suggest that an increase in lung volume is due to the development of an optimized respiratory movement pattern because of higher change in the abdominal area. Hydrostatic pressure acting on the swimmer’s body increases respiratory work which, after long-term training, contributes to the increase in muscle strength [17]. Other authors suggest that the lungs of competitive swimmers are characterized by higher lung capacity [18] and diffusion capacities [19,20] in comparison with a healthy control group. Meanwhile, Bovard’s research (2018) results show that competitive swim training does not influence the development of lung size or function [21]. Also, in some studies, it has been shown that IMT benefits were the result of a dose-dependent effect of swim training [22,23,24].

Previous studies have shown that training of respiratory muscles improves athletic performance in some disciplines, e.g., cycling, rowing, and running [25,26,27], and may lead to changes in the functional parameters of the respiratory system [28].

As confirmed by the literature, the form of additional IMT is also applicable to swimmers [23].

In the available literature, one can find a few studies on the use of IMT in disabled athletes who practice sport professionally. These studies concern basketball players on wheelchairs with spinal cord injuries. People with disabilities are often characterized by abnormalities in the functioning of the respiratory system due to changes in their movement system. Therefore, it seems appropriate to add breathing training to strengthen the respiratory muscles and improve the ventilation of these athletes [29,30]. Additionally, it has been shown that that the respiratory system can be a limiting factor in the aerobic performance of highly trained athletes [31]. The reasons for this could be increased respiratory work, respiratory muscle fatigue, and dyspnea [32]. Inspiratory muscular training (IMT) has been widely implemented in several clinical situations, for example, chronic obstructive pulmonary disease (COPD), postoperative coronary artery bypass surgery, children with Duchenne muscular dystrophy, and post-bariatric surgery [33]. The studies of swimmers that have been described were carried out on a small group of competitors, and the obtained results were often different [34]. Therefore, the aim of this study was to assess the efficacy of 8-week IMT for disabled swimmers, when added to standard sports training, on selected parameters of lung ventilation and the functioning of respiratory muscles.

## 2. Materials and Methods

### 2.1. Subjects

This study included 16 disabled swimming division athletes from Wroclaw’s ‘Start’ Sports Association for the Disabled. The following factors were taken into account in the selection of competitors: age, professional training experience, and sports level (Table 1). Each of the athletes provided written consent to participate in the research. This research received permission from the ethics committee no. 17/2019. Swimming classes have been granted by the classifier International Paralympic Committee. On the basis of the type of disability, the trainer selected appropriated training categories: basic strength training—EN1; threshold strength training—EN2; stress overload training—EN3, and lactic tolerance training—SP1; lactic training—SP2; sprint training—SP3 [35].

**Figure 1:**
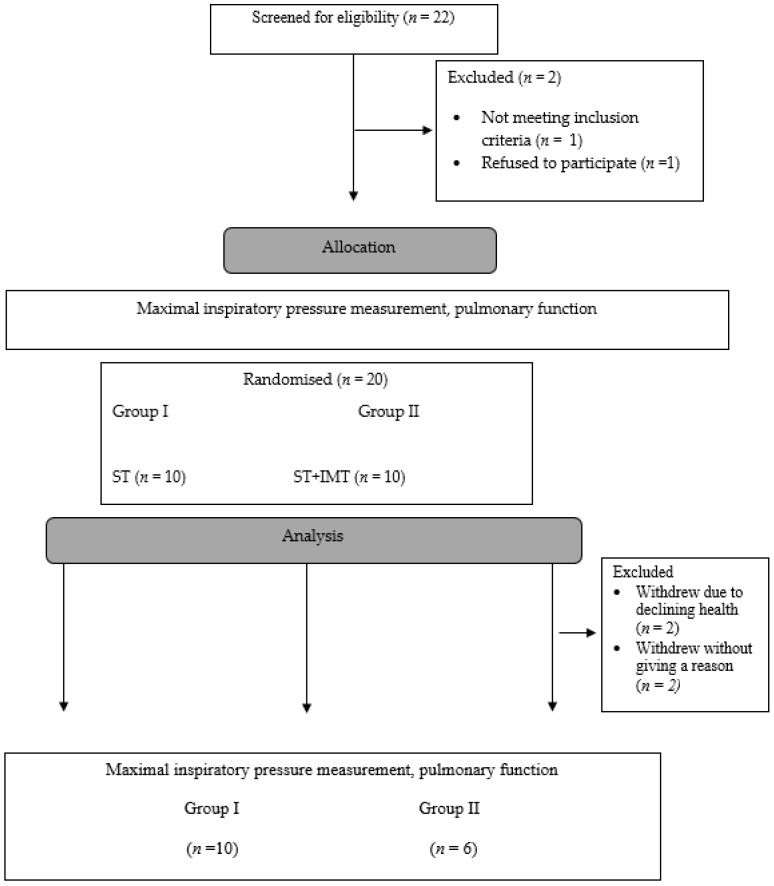
Design and flow of participants throughout the study. ST—swimming training; IMT—inspiratory muscular training.

The subjects were divided into two groups: ST consisted of 10 athletes performing swimming training (ST), IMT consisted of 6 competitors additionally performing IMT on the Threshold IMT device from Respironics (Figure 1). The selection for groups was random (according to the distribution table).

### 2.2. Experimental Procedures

To qualify for the study, participants had to meet the following inclusion criteria: age 16–20; a minimum of 8 years training experience, and not using another respiratory therapy. After qualifying for the tests, each participant was familiarized regarding all the experimental procedures. Before commencement of the study sessions, the competitors were subjected to anthropometric tests and functional respiratory system tests. Assessment of the respiratory system was carried out using FlowScreen (780,578, version 1.3, Viasys Healthcare, Jaeger, Hoechberg, Germany).

The research was in accordance with the criteria of the American Thoracic Society/European Respiratory Society from 1993 [36]. As a result of spirometry tests, a flow–volume curve was derived, and this procedure was repeated three times, with an expiration time that lasted for a minimum of 6 seconds, and at the measurements of at least two of the three trials had to be reproducible; the forced vital capacity (FVC) and forced expiratory volume in first second (FEV1) parameters did not differ by more than 5% [36].

The following parameters were analyzed: vital capacity (VC, liters), forced vital capacity (FVC, liters), forced expiratory volume in first second (FEV1, liters), and peak expiratory flow (PEF, liters/seconds). Then, % predicted values were calculated and reported for all spirometric measures [37].

The maximum inspiratory pressure (PImax), expressed in cmH_2_O, and measurement of maximum expiratory pressure (PEmax), expressed in cmH_2_O, were used to assess the strength of the respiratory muscles.

In order to assess the occurrence of lung ventilation disorders, a criterion was applied where FVC and FEV1 values equal to 75% and above were considered as the norms. Values below 75% FVC were considered to be restrictive-type lung ventilation disorders, and values below 75% of FEV1 were assessed as obstructive ventilation disorders [38].

An assessment criterion produced in accordance with the ATS/ERS—Statement on Respiratory Muscle Testing was used to assess the occurrence of inspiratory muscle strength disorders. PImax greater than or equal to 80 cmH_2_O was considered to be the correct value [39].

### 2.3. Swimming Training

Due to the high sporting level of the competitors and the range of disabilities in terms of their degree and type, the training of athletes took an individual form. During one training unit, as well as throughout the training cycle, the competitors swam different distances, which resulted from their functional abilities depending on the type and degree of disability (the greater the disability, the shorter the distance covered).

Training took place 8 times a week in a 25 m swimming pool. Additionally, stretching and strength exercises were conducted in the training hall three times a week. In the first mesocycle, the average training volume covered a specific distance. Table 2 shows the training volume in the three mesocycles during which the examination was carried out. The training volume and time in each zone were the same in both groups.

In the last two weeks before the main competition (i.e., the period after the second test), the athletes trained over smaller distances, focusing mainly on the improvement of technical and speed elements.

### 2.4. Respiratory Training

IMT was carried out on an 8-week cycle, 5 times a week, 2 times a day (1 training session in the morning and 1 session in the evening). The inhale should be full, fast, and strong, and the exhale long and slow. It is important that each inhale begins with the residual volume (RV), i.e., after a deep exhalation [40]. The competitors performed one training session a week under the supervision of a physiotherapist at the swimming pool of the University School of Physical Education in Wroclaw before the regular swimming training. The rest of the workouts during the week were performed at home. Each participant took part in instructional classes determining the proper technique of the exercises on the Threshold IMT equipment from Philips Respironics. The prescribed workouts were performed in a standing position.

The athletes performed the prescribed training individually on personal devices. After determining each athlete’s PImax, the training load was set individually. The assumption of the used IMT training indicates the use of the PImax output value in the load of the entire training cycle. The load increased in accordance with the given diagram in Table 3. The initial load that was determined ensured the safety of the training to be performed [41].

During training, the athlete should not have experienced discomfort, shortness of breath, or pain [28]. All participants in the IMT group fill out a training diary, as a control, to assess adherence to training sessions.

### 2.5. Statistical Analysis

The results were analyzed using the program Statistica version 7.0 by StatSoft from Dell (Round Rock, TX, USA). The Kolmogorov– Smirnov test and the Lilliefors test were used to assess the normality of distribution. Basic descriptive statistics were calculated.

In order to show the differences between the groups that were separated based on somatic parameters, a two-tailed Student’s *t*-test was used (the distribution of these characteristics was normal). The parameters of the respiratory system between the groups and the tests were evaluated using repeated measures ANOVA analysis and Duncan’s post hoc test. The differences at *p* < 0.05 were considered significant. Effect sizes for the magnitude of statistically significant group differences were calculated using the Cohen’s d statistic, and effect sizes were expressed as small (>0.20), moderate (between 0.50 and 0.80), and large (>0.80). The equation for used Cohen’s $d=\frac{\left(M2-M1\right)}{SDpooled}$, where $SD_{pooled}=\sqrt{\frac{\left(SD12+SD22\right)}{2}}$.

## 3. Results

The analysis of test results began from the presented descriptive data of the respiratory functional parameters and the inspiratory and expiratory muscle strength in the groups (Table 4). *p*-values are given in Table 5.

The use of swimming training alone resulted in a significant increase only in lung capacity. However, in the group with additional IMT, there was a significant increase in all studied respiratory system functional parameters. A significant increase in the strength parameters of respiratory muscles was observed in both the ST and IMT group. However, after 8 weeks, significantly higher values of all parameters were observed in the group of athletes with additional IMT. (Table 5).

The analysis of results also assessed the frequency of lung ventilation disorders in the examined groups. After the training programs in ST, an improvement was achieved in 10% of people with restrictive-type disorders. However, after the training programs were applied to the ST group, there was no reduction in the occurrence of obstructive disorders. In the IMT group, a regression in lung ventilation disorders was achieved (Table 6).

In evaluating the inspiratory muscle strength based on the PImax value, it was found that all persons in the ST group and 83.3% in the IMT group of subjects had reduced strength in these muscles. In most of the swimmers, the regression of inspiratory muscle strength disorders, after swimming training, was achieved in the IMT group (Table 7).

## 4. Discussion

Addition of inspiratory muscle training in the standard swim training of disabled swimmers resulted in a significant increase in all lung function parameters and inspiratory muscle strength (Appendix A).

The subject of disabled swimmers is rarely researched. There are also only a few studies assessing the strength of respiratory muscles of healthy people who practice swimming.

Sable et al. (2012) found that swimming training improves lung volumes because the respiratory muscles, including the diaphragm, overcome the increased pressure exerted by water during the breathing cycle. The authors also believe that this may lead to functional improvement of respiratory muscles, as well as changes in the lung and chest wall elasticity [42]. The results of our research also confirm this. The swimming training undertaken by the disabled athletes caused significant improvements in the strength of their inspiratory and expiratory muscles. Intercostales interni muscles, which are generally considered muscles of expiration, also function in inspiration during forced ventilation [43]. In our study, deep expiration that reached the approximate RV may have been promoted when changing PEmax during IMT.

Clanton et al. [44] found that swim training increases VC, TLC, and FRC with no effect on RV. Other reports of the mechanisms relating to the large lung volumes of swimmers with anthropometric data include Armour’s research (1993). Swimmers had significantly increased total lung capacity, vital capacity, inspiratory capacity, and forced expiratory volume in one second (FEV1). These studies suggest that swimmers may have achieved greater lung volumes than other participants because of developing physically wider chests that contain an increased number of alveoli [19]. Some authors debate whether this is the result of genetic determinants [45] and/or an adaptation to swim training [46].

The positive effects of swimming on lung function have also been confirmed by Lazovic-Popovic et al. (2015) who, in their studies, assessed the function of the respiratory system and its correlation with anthropometric traits in swimmers, soccer players, and non-athletes. They noticed that the swimmers were characterized by statistically higher values of volumetric parameters of the respiratory system (VC, FVC, FEV1 and FEV1/FVC) in comparison to the other subjects [47]. The study by Wells et al. confirmed this tendency [48]. In addition, Doherty and Dimitriou (1997) found that swimmers had a higher forced expiratory volume in the first second (FEV1) regardless of age and body height, compared to athletes practicing sports under land-based exercise, such as running, and those leading a sedentary lifestyle [49]. Furthermore, swimmers were found to have better physiological adaptation [50]. Cordain et al. (1990) showed that swimmers had significantly higher values for all static lung volumes when compared to other participants, in spite of the fact that all subjects were matched for height and age. At the same time, research results suggest that swimming training does not cause increases in maximal static inspiratory and expiratory pressures [51,52].

In our research, an observation was made regarding the influence of both swimming training and swimming training with added inspiratory muscle training on lung ventilation parameters. In the group with swimming training only, a significant increase in the vital capacity of the lungs was observed, while a significant increase in all ventilation parameters (VC, FVC, FEV1, PEF) was only observed in the group in which IMT was additionally used. This indicates the importance of using this type of training in the process of sports education, and even more so since existing disorders of lung ventilation and inspiratory muscles decreased as a result.

Lemaitre et al. (2013) also assessed the effects of IMT on the function of respiratory muscles and swimming performance. They demonstrated that the inclusion of this type of training resulted in greater effectiveness than swimming training alone. This study utilized respiratory muscle endurance training, which is similar to threshold IMT but is not exactly the same [53]. In respiratory muscle endurance training, voluntary normocapnic hyperpnea is allowed, without the limitation of lower limb muscle involvement [54].

The results of our research also showed a highly significant increase in the maximum inspiratory pressure following 8 weeks of additionally conducted IMT in disabled swimmers compared to the group that only had swimming training.

There are very few studies evaluating the use of IMT in disabled athletes. They mainly concern people in wheelchairs with spinal cord injuries. Such studies were conducted by Litchke et al. (2008), who evaluated 10 weeks of IMT on people in wheelchairs with spinal cord injuries who were participating in basketball training. They divided the subjects into two study and control groups, with each of them taking into account the level of physical activity based on the number of hours of participation in sports activities. Researchers showed a significant difference between groups in terms of the change in maximum inspiratory pressure. In the group with respiratory muscle training, the results were significantly higher [30]. This is also confirmed by the results of our research involving disabled swimmers. Our research also shows the same changes: In the ST group, PImax increased by 27.57 % and by 34.92% in the IMT group.

Goosey-Tolfrey et al. (2010) assessed the effects of inspiratory muscle training on the functions and on the repeatability of sprint velocity in disabled basketball players. In the group with proper IMT training, they noted an increase of 17% in the maximum inspiratory pressure and an increase of 23% in the maximum exhalation pressure. Interestingly, improvements were also found in the IMT placebo group, where the maximum inspiratory pressure increased by 23% and the maximum exhalation pressure by 33% [29]. Our study did not include the group that completed the IMT placebo. Currently, there are several IMT placebo protocols, but it has been shown that they have no effect on the functioning of the lungs and respiratory muscles [55]. The results of our research confirm the tendency of change in maximum inspiratory pressure. In our research, in the group where IMT was additionally used, the maximum inspiratory pressure parameter increased by 34.92% and the expiratory pressure parameter increased by 19.94%. In the group without IMT, however, inspiratory pressure increased by 27.57%, and maximum exhalation pressure by 19.97%.

According to Lomax, 6 weeks of pressure threshold IMT significantly increased PImax in well-trained youth swimmers, although this did not automatically translate into improved swimming time trial performance. The training protocol should be a combination swim training and inspiratory muscle training [22].

At the same time, there are reports indicating that there may be a dose–response relationship depending on swimming training and its impact on the functioning of the lungs and respiratory muscles. Mickleborough et al. (2008) have demonstrated that a rigorous 12-week competitive swimming training program improves pulmonary function and increases respiratory muscle strength and endurance in elite swimmers to the same extent as a 12-week combined IMT and competitive swimming training program. Moreover, it has been shown that the addition of IMT to swim training was not beneficial for high swimming training loads [23]. Interestingly, Shei et al. (2016) have shown that swimmers with a markedly lower training volume and intensity have additional benefits from combining flow-resistive inspiratory muscle training and swim training [24].

## 5. Conclusions

1. Prior to the commencement of training, lung ventilation disorders were found to be at similar levels in both the restrictive and obstructive domains among all disabled swimmers. In both groups, a reduction in the frequency of disorders was achieved following the applied training, exhibiting higher levels in the group that had implemented IMT, where regression in both types of disorders was achieved.
2. Before the start of the tests, the strength of inspiratory muscles was reduced in the majority of swimmers. Swimming training resulted in a slight improvement, while the inclusion of additional IMT significantly improved muscle strength in more than half of the respondents.
3. The inclusion of IMT is an important element complementing swimming training, allowing for greater increases in lung ventilation parameters and the strength of respiratory muscles in the disabled swimmers.

## Figures and Tables

**Table 1:** Characteristics of the athletes in each group.

Parameters	Distribution	ST	IMT
**Age (years)**		18.20 ± 4.64	18.50 ± 4.97
*p*		0.55	
**Height (m)** Mean ± SD		1.69 ± 0.09	1.70 ± 0.07
*p*		0.96	
**Body mass (kg)**		58.90 ± 9.15	60.80 ± 5.95
*p*		0.67	
**BMI (kg/m^2^)** Mean ± SD		20.37 ± 1.69	20.99 ± 1.30
*p*		0.42	
**Sex**	Women	5	3
	Men	5	3
**Training experience (years)**		9.70 ± 2.51	10.10 ± 2.77
*p*		0.07	
**Sporting achievements**	Polish Championships	8	6
	Polish Junior Championships	9	5
	European Championships	0	1
	Paralympics	1	1
**Type of dysfunction**	Motor system	6	3
	Eye	2	2
	MDP syndrome	2	1
**Swimming class**	S5	1	0
	S7	1	1
	S8	3	0
	S9	3	2
	S10	2	3

BMI—body mass index; MDP syndrome—cerebral palsy (*paralysis cerebralis infantium*); Swimming class: S—freestyle, butterfly, and backstroke events; 1–10—sport classes for athletes with physical impairment. Swimming classes have been granted by the classifier International Paralympic Committee.

**Table 2:** Training volume.

Variable	First Mesocycle	Second Mesocycle	Third Mesocycle
**distance (m)**	4750 ± 353.55	4000 ± 353.55	2750 ± 353.55
**swimming volumes (%)**			
**REC**	30% (1425 ± 106.07 m)	20% (800 ± 141.42 m)	20% (550 ± 70.71 m)
**EN1**	30% (1425 ± 106.07 m)	30% (1200 ± 212.13 m)	30% (825 ± 106.07 m)
**EN2**	30% (1425 ± 106.07 m)	30% (1200 ± 212.13 m)	30% (825 ± 106.07 m)
**EN3**	5% (237.5 ± 17.68 m)	10% (400 ± 70.71 m)	5% (137.5 ± 17.68 m)
**SP1 and SP2**	5% (237.5 ± 17.68 m)	10% (400 ± 70.71 m)	15% (412.5 ± 53.03 m)

m—meters; REC—recovery zone; EN1—basic strength training; EN2—threshold strength training; EN3—stress overload training; SP1—lactic tolerance training; SP2—lactic training; (Mean ± SD).

**Table 3:** Inspiratory muscle training.

Week	1	2	3	4	5	6	7	8
**Load (% PI_max_)**	30	40	40	50	50	60	60	60
**Time (min)**	2 × 5	2 × 8	2 × 11	2 × 11	2 × 13	2 × 13	2 × 15	2 × 15
**One session (breaths)**	30	30	30	30	30	30	30	30

**Table 4:** Mean values, standard deviations, and the increase in ventilator parameters and PImax, PEmax in the examined groups.

Variable	ST Group				IMT Group			
	Test I (1)	Test II (2)	Effect Size	Change (%)	Test I (3)	Test II (4)	Effect Size	Change (%)
	Mean ± SD	Mean ± SD			Mean ± SD	Mean ± SD		
VC (l)	3.33 ± 0.81	3.78 ± 0.81	0.55	13.51	4.08 ± 0.94	4.69 ± 0.9	0.91	14.95
VC%	80.69 ± 12.24	91.46 ± 13.11	0.84	13.35	93.33 ± 25.1	112.5 ± 23.54	0.9	20.54
FVC (l)	3.38 ± 0.97	3.74 ± 0.96	0.37	10.65	3.78 ± 1.17	4.82 ± 0.95	0.97	27.51
FVC%	81.08 ± 12.73	90.53 ± 12.84	0.73	11.65	92.33 ± 32.77	117.33 ± 25.75	0.84	27.07
FEV_1_ (l)	2.86 ± 0.98	3.124 ± 1.01	0.26	9.23	3.02 ± 1.29	4.05 ± 0.71	0.98	34.1
FEV 1%	82.43 ± 19.92	89.57 ± 21.5	0.50	8.66	81.78 ± 39.51	105.68 ± 39.5	0.85	29.22
PEF (L/s)	5.19 ± 2.03	5.73 ± 1.87	0.27	10.4	5.51 ± 3.11	7.49 ± 1.34	0.82	35.93
PEF%	69.83 ± 21.78	76.97 ± 20.55	0.33	10.22	73.83 ± 43.95	98.33 ± 20.8	0.78	33.18
PImax (cmH_2_O)	41.0 ± 22.6	56.6 ± 2.42	0.66	27.57	61.44 ± 19.72	94.38 ± 23.48	2.35	34.92
PImax%	57.72 ± 28.38	79.54 ± 30.83	0.73	21.82	88.33 ± 29.42	133.33 ± 31.49	1.47	33.75
PEmax (cmH2O)	60.97 ± 30.89	76.13 ± 26.96	0.52	19.97	88.52 ± 23.75	110.5 ± 21.81	0.96	19.94

Abbreviations: VC—vital capacity; FVC—forced vital capacity; FEV_1_—forced expiratory volume in first second; PEF—peak expiratory flow; PImax—maximum inspiratory pressure; PEmax—maximum inspiratory pressure.

**Table 5:** *p*-values for Duncan’s post hoc test for selected ventilation parameters and PImax and PEmax.

Variable	I–II in ST Group (1–2)	I–II in IMT Group (3–4)	I in ST Group and Test I in IMT Group (1–3)	II in Group ST and Test II in Group IMT (2–4)
VC (l)	**0.02** *	**0.01** *	0.44	0.29
VC %	**0.01** *	**0.01** *	0.34	0.21
FVC (l)	0.55	**0.04** *	0.89	0.27
FVC %	0.49	**0.04** *	0.77	0.13
FEV_1_ (l)	0.71	**0.02** *	0.99	0.41
FEV 1%	0.72	**0.02** *	0.99	0.36
PEF (L/s)	0.73	**0.05** *	0.99	0.49
PEF %	0.68	**0.04** *	0.99	0.52
PImax (cmH_2_O)	**0.02** *	**0.00** *	0.43	**0.04** *
Pimax %	**0.02** *	**0.00** *	0.31	**0.02** *
PEmax (cmH_2_O)	**0.04** *	**0.02** *	0.32	0.16

Abbreviations: VC—vital capacity; FVC—forced vital capacity; FEV_1_—forced expiratory volume in first second; PEF—peak expiratory flow; PImax—Maximum inspiratory pressure; * statistically significant inter-testing session or intergroup difference at the *p* ≤ 0.05 level.

**Table 6:** Occurrence of restrictive and obstructive pulmonary ventilation disorders in the examined groups.

Group	Restrictive Disorders before Training (%)	Restrictive Disorders after Training (%)	Obstructive Disorders before Training (%)	Obstructive Disorders after Training (%)
**ST**	30	20	20	20
**IMT**	33.3	0	33.3	0

**Table 7:** The incidence of maximum inspiratory pressure disorders (PImax).

Group	Before Training	After Training
	(%) Athletes below the Norm	(%) Athletes below the Norm
**ST**	100	80
**IMT**	83.35	16.7

## Appendix A

### Appendix A.1. Perspectives:

The introduction of inspiratory muscle training to a typical training cycle for disabled swimmers is innovative and, so far, no research reports on this subject have been found, and its use is safe and requires only inexpensive equipment. Our results confirmed that the use of IMT resulted in a complete reduction of lung ventilation disorders (obstruction and limitation) in people with disabilities. The addition of IMT supplemented swimming training, resulting in a greater increase in lung ventilation parameters and the strength of respiratory muscles in disabled swimmers. It can also be assumed that the use of IMT may also contribute to achieving better sports results by improving lung ventilation and, also, in improving physical performance [56]. Such confirmations were presented by other authors who assessed the impact of 6-week IMT training on the measurement results of 100, 200, and 400 m crossing time in healthy swimmers, with improved distances [57].

The new findings can be summarized as follows:

1. There have previously been no research reports on ways to use inspiratory muscle training (IMT) that were easy and safe.
2. The use of IMT led to a complete disappearance of lung ventilation disorders (the obstruction and restriction) in disabled swimmers.
3. The inclusion of IMT supplemented the swimming training, resulting in greater increases in lung ventilation parameters and the strength of respiratory muscles in disabled swimmers.

### Appendix A.2. Limitations:

This was a small-scale trial; therefore, generalizability of the results to clinical practice is limited. Thus, verification of the long-term effects using a larger sample size and a longer follow-up period should be considered in further studies. It is also necessary to verify the influence of pulmonary volumes on athletes’ performance. Moreover, another limitation is that no performance measures were employed so, at present, it is unknown whether the observed enhancements in pulmonary function would translate to ergogenic effects. All athletes from the sports club were included; hence, represent a convenience sample. Future studies should consider increasing the group by including other sports clubs.
